# Emerging Technology: Preparing Tomorrow's MCH Workforce to Innovate for Equity

**DOI:** 10.1007/s10995-021-03371-5

**Published:** 2022-01-20

**Authors:** Marissa McKool, Sarah Han, Jaspal Sandhu, Cassondra Marshall, Sylvia Guendelman, Kim Harley

**Affiliations:** Wallace Center for MCAH, UC Berkeley School of Public Health, 2121 Berkeley Way, Berkeley, CA USA

**Keywords:** MCH, Technology, Innovation, Workforce development, Training pipeline

## Abstract

**Purpose:**

This commentary proposes a new direction to train the MCH workforce by leveraging today’s rapidly changing innovation and technology to address persistent health inequities.

**Description:**

We outline the creation of an MCH technology and innovation training pipeline developed by harnessing creative funding opportunities, diversifying training modalities, and expanding partnerships beyond traditional academic-practice partners, that be replicated and adapted by other academic programs.

**Assessment:**

Technology and innovation will continue to be a growing intersection between health and equity, and we must create a robust pipeline of MCH leaders prepared to collaborate with entrepreneurial and innovation leaders.

**Conclusion:**

Technology offers an important opportunity to improve MCH outcomes and reduce disparities, but only if we train the MCH workforce to seize these opportunities.

## Significance Statement

The landscape of public health and healthcare is rapidly evolving, yet the U.S. continues to face racial disparities in maternal and infant health. The adoption of technology in our daily lives has grown exponentially, especially as a result of the COVID-19 pandemic. Research shows that technology can help reduce barriers to healthcare and provide key insights into health information. However, a critical understanding of how to leverage today’s fast growing technology into a new digital age for public health is lacking from current MCH training. This commentary proposes a new method of developing a training pipeline of leaders in MCH technology and innovation to address health equity.

## Introduction

The public health landscape is rapidly evolving as we reckon with deepening social inequities, burgeoning digital technologies, and the global COVID-19 pandemic. Despite important gains, the U.S. continues to face persistent racial disparities in maternal and infant health that have repercussions across the lifespan for individuals, families, health systems, and communities (Holdt Somer et al., 2017; Howell, 2018; Lu, 2018). Meanwhile, adoption of technology in healthcare and research has grown exponentially, through the development of health informatics and electronic health records (EHRs) that allow for improved efficiency and opportunities for data sharing, implementation of telehealth modalities that promote communication between health care providers and patients, search engines (e.g., Google) and social media that make health information more widely accessible, and increased use of smartphones that allow for connectivity between health care consumers and providers (Barclay et al., 2014; McNabb et al., 2006). The use of healthcare technology, such as telehealth and remote patient monitoring, has further expanded during the COVID-19 pandemic in part because of updates in telehealth regulations (Webster, 2020). The pandemic also revealed major gaps in the maternal-child safety net and exposed how years of community disinvestment and systemic racism have exacerbated health inequities (McCloskey et al., 2021).

As we examine the skills that the emerging workforce will need, a critical understanding of technology and innovation and their growing role in public health—as well as competence in applying emerging digital strategies—are vital for future MCH practitioners, researchers, and advocates (Czabanowska & Kuhlmann, 2021; Lu, 2019; Shah et al., 2020; Streeter, 2015). The MCH workforce of tomorrow needs to be equipped to leverage this new digital landscape to address persistent health issues and unlock opportunities to achieve MCH equity. In this paper, we highlight how the Wallace Center for Maternal, Child, and Adolescent Health at UC Berkeley is engaging and training students at the nexus of technology, innovation, and MCH to be the next generation of public health leaders.

## The Wallace Center for Maternal, Child, and Adolescent Health

The Wallace Center for Maternal, Child, and Adolescent Health (MCAH) is a multidisciplinary research and training center established in 2015 with an endowment from Helen Wallace, the first Chair of the MCH program at UC Berkeley’s School of Public Health. In the formative development of the center, one of the authors (S.G.) consulted with MCH leaders, faculty, and other key stakeholders across the country to identify current research and training gaps. Stakeholders agreed that tremendous opportunities exist to understand the value and effectiveness of various technologies directed at diverse MCAH populations. This would require engaging the MCAH communities and populations to continuously assess their interest and actual use of technologies, their preferences, satisfaction and solutions to problems that these technologies present.

Our Center’s mission is to conduct actionable research in innovation and technology to advance MCH and reduce inequities, while engaging community and training future leaders. In 2020, we created a new training pipeline approach in MCH technology and innovation that focused on three defined areas of technology and innovation—(1) Integrating technology into services; (2) Using technology to gain new research insights; and (3) Redefining innovation in public health.

## Creating a Training Pipeline in MCH Technology and Innovation

To train future public health leaders in these three specific areas of MCH technology and innovation, we expanded our interdisciplinary partnership approach, developed creative funding mechanisms, and placed trainees on projects with cutting-edge research methodologies. We intentionally included our Wallace Center faculty members in our training pipeline, all of whom are public health faculty in community health science, supporting them as they expand their own expertise to become principal investigators on digital research initiatives and instructors of new and innovative courses within their topic areas of expertise. Undergraduate, masters, doctoral, and post-doctoral students in public health programs, or technology-focused disciplines (e.g., engineering) with a strong public health interest, obtained new knowledge and skills through innovative courses, research positions, internships, dissertation awards, and fellowships. We strategically designed our training opportunities to include students and faculty from both public health and technology-focused disciplines (e.g., engineering) to foster cross-sector education in MCH.

We leveraged existing partnerships and started new collaborations with diverse stakeholders across technology, healthcare, innovation, and public health. To develop new partnerships, we leveraged the strong relationships of our Dean with technology and healthcare leaders, utilized our expansive alumni base to identify unique project opportunities, and connected with internship host sites to explore collaboration possibilities. Partnerships also included research and teaching collaborations with campus faculty outside of public health in technology-focused departments (e.g., data science).

We used three creative funding mechanisms to provide training opportunities (Table 1). First, we invested seed funding from our center’s endowment to support student research positions on five Wallace faculty-led pilot projects. The funding support varied by project need and ranged from $5000 to $50,000 per project. This seed funding functioned as a grant and differed from departmental funding as they incentivize faculty to incorporate innovation and technology into their research. Second, we created a summer internship placement program for Master of Public Health (MPH) students through a matched funding model. The Wallace Center and a host organization co-funded a student position to work on a technology or innovation-based project, resulting in more student internship opportunities. In the past two years, eleven MPH students completed their internship at eight different organizations ranging from private consulting firms to healthcare providers. Students were paid $10,000 for their three-month, full-time placement. Third, our faculty members secured a training grant from our campus to create and implement an applied research undergraduate course in MCH infodemiology, a research method to assess online search-term information to inform public health (Guendelman et al., 2020; Zepecki et al., 2020). This grant provided funding for a year-long course and one graduate student instructor. To increase support for the applied training, the Wallace Center provided funding for an additional student instructor. The purpose of these projects are to advance MCH research and training opportunities, with the potential to secure additional external research grants.

**Table 1:** Wallace investments in research and training, May 2020–August 2021

Funding model	Core activity	Wallace investment range	No. of investments	Catalyzed funding
Seed funding	Applied research	$5000–$50,000	5	Ongoing external research grants
Internship matching	Field training	$5000–$10,000	11	Matched funding from 6 host organizations^a^
External grants	Academic training	$14,000	1	Funding from campus grant

^a^Three host organizations had more than one student placement. Placements at two community-based organizations were fully funded by the Wallace Center due to the organization’s limited financial resources

Below, we describe the three defined areas of technology and innovation and highlight a specific training opportunity provided by the Wallace Center for each area (see Table 2. for an expanded list of examples). The Wallace Center takes a life course approach to MCH research and the projects described here focus on maternal, reproductive health, and family planning.

**Table 2:** MCH Applied Research Training Opportunities Across Three Categories of Innovation

Topic area	Project(s)	Trainee	Partner
Integrating technology into care			
Telehealth	Assessing facilitators and barriers to telehealth use during the COVID-19 pandemic among maternal fetal medicine providers	MCH MPH student	Stanford School of Medicine
Remote patient monitoring (RPM)	Patient acceptability study for postpartum at home blood pressure RPM for patients with hypertensive disorders of pregnancy	MCH MPH student	County Hospital
Using technology to gain new insights			
Qualitative data from social media	Assessing Reddit’s utility to conduct family planning and reproductive health research through machine learning methodologies	Social welfare PhD student	
Applying infodemiology principles to search engine data	Using Google Applied Program Interfaces (APIs) to characterize internet searches related to family planning, reproductive health, nutrition, mental health, and youth homelessness	DrPH student Undergraduate PH students Undergraduate data science students	School of Information, Electrical Engineering and Computer Science Department, Data Science Initiative, Bixby Center
Big data from mobile health apps	Leveraging existing data from large health tracking apps and launching user surveys, to study diverse and hard-to-reach populations and research understudied health outcomes such as menstruation	MCH MPH Student Electrical engineering and computer science PhD student	Leading mHealth App company with a commitment to data privacy, ethics, and research
Defining innovation in public health			
Community-based care	Leading strategic planning, implementation science, and evaluation projects to set a research agenda on community-doula care and to provide evidence to inform intervention strategy	MCH MPH student	Community doulas, doula clients, researchers, clinicians, policy advocates, payers
Imaging alternative futures	Blending futures thinking, racial equity, and human-centered design to imagine alternative futures for maternal health and the role of emerging technology, from the doula perspective	Undergraduate student MCH MPH student	Doulas, School of Social Welfare
Discrete choice experiment	Developing a digital survey tool using a discrete choice experience to elicit real-time adolescent preferences for contraceptive methods and services that can be used in the absence of revealed preference data in clinical settings	DrPH student	

This table does include projects from the MPH summer internship placement program

### Integrating Technology into Services

An expanded integration of technology in care has the potential to address the social determinants of health contributing to health disparities by improving access and reducing barriers to healthcare and other wrap around services. Telehealth services can help extend healthcare to rural areas and underserved urban communities by reducing challenges around transportation, childcare, and work schedules (Hoffman, 2020; Meyer et al., 2020). The use of remote monitoring tools, such as wearable health devices or mobile tracking apps, can improve at-home management of chronic health conditions through the collection of real-time data (Fischer & Kleen, 2021; Milne-Ives et al., 2020; Vaghefi & Tulu, 2019).

We advised on the design of a text-based blood pressure monitoring intervention for patients with hypertensive disorders of pregnancy for a county hospital in Oakland, CA delivering nearly 1000 births in California’s Medicaid program per year. A UC Berkeley MPH student served as a graduate student researcher on the project in Spring 2021. By conducting the initial literature review for the project and working with clinicians, the student developed unique expertise in telehealth and remote patient monitoring (RPM) research evidence that otherwise would not have been obtained in their coursework. The OB/GYN provider on the project and Wallace faculty advisor (K.H.) also expanded their understanding and knowledge of perinatal telehealth and RPM by learning from the student. In addition, the student worked closely with the OB/GYN provider to design the study and learned about the challenges, opportunities, and complexities of implementing innovative and technology-based interventions in publicly funded healthcare systems. The student has since graduated and secured a full-time position conducting academic research on digital health and health systems.

### Using Technology to Gain New Insights

Traditional epidemiologic research relies on enrollment, surveying, and follow-up of individual study participants, which can be resource heavy. Millions of consumers now provide real-time quantitative health data, also known as “big data,” into mobile apps and other devices that can be longitudinal, quickly synchronized, and are less likely to suffer from recall bias, social desirability bias, or mistakes occurring during manual data transfers (Fischer & Kleen, 2021). Big data also encompass qualitative data from social media sites such as Reddit, Facebook, and Twitter that can be used to conduct digital ethnographies or infodemiology analyses about public health issues (Eysenbach, 2009; von Hippel, 2018).

One of the authors (C.M.) is a family planning researcher and is assessing the value of social media and online community data for contraception research by conducting a qualitative analysis of a birth control Reddit community. This work utilizes a novel machine learning-enabled approach to analyze user-generated big data from digital communication platforms (von Hippel, 2018). In Spring 2021, a doctoral student from the School of Social Welfare with a designated emphasis in computational data science and engineering joined the research team. The student’s expertise in data science enabled the faculty to gain a deeper understanding of the use and limitations of machine learning methodologies for MCH research. Machine learning describes a set of techniques that run analytical models by adapting to data patterns (Mooney & Pejaver, 2018). By conducting the analysis of user-generated comments, the student gained a deep understanding of family planning issues, such as lack of access to contraceptives, experienced by Reddit users. The student also received education and mentorship on MCH equity issues and public health research ethics that likely would not have been gained in their program coursework or other research opportunities. As a result of this project, the student is now working on a manuscript outlining their findings and applying other machine learning algorithms to the data.

### Defining Innovation in Public Health

Leveraging human-centered design approaches has strong potential for developing novel interventions to improve MCH (Vechakul et al., 2015). Human-centered design is an innovative strategy that involves first cultivating deep empathy with users and understanding their needs before defining the problem at hand. The next steps are to ideate solutions and then prototype and test them in creative, interactive ways in direct collaboration with the users (Lister et al., 2017). This ensures that the resulting solution or product is engaging, adaptive, and relevant to the specific user group it was designed for.

We are blending futures thinking, racial equity, and human-centered design to imagine alternative futures for maternal health and the role of emerging technology by gathering doula perspectives for the future of maternal health care. The project team consisted of a faculty lead (J.S.), who has a background in engineering and design thinking, and an advisory board of faculty experts in qualitative doula research and practicing community doulas. The research team included an undergraduate public health student, an MCH MPH student, and a Boston University MCH student between Spring and Summer 2021. These students gained insights into human-centered design methodologies, as well as traditional qualitative research. In addition, the advisory board members broadened their understanding of design thinking principles and methodologies from the core research team and the faculty lead deepened his understanding of entrenched issues in MCH equity and maternal health care. In the Fall 2021, students will be holding design thinking sessions with the project partners and doulas.

## Opportunities to Innovate MCH Training

The MCH perspective is a critical but largely unheard voice for the prioritization and development of innovative and technological solutions to entrenched public health problems. The MCH workforce is poised to center equity and engage hard-to-reach and underrepresented populations, outline novel ethical research methodologies, and build a comprehensive evidence-base for interventions (digital and otherwise). If we do not train our MCH workforce in the space of innovation and technology, we risk losing opportunities in the development and dissemination of cutting-edge research and interventions to address entrenched MCH issues. Given the rapid evolution of technology, we must be agile in leveraging innovative and digital solutions for public health research and practice to advance health equity. We need trained MCH professionals at the table, alongside experts and decision-makers in business, medicine, data and computer science, and other technology sectors.

Developing and implementing dynamic, high-quality, engaging, and diverse training opportunities that incorporate technological innovations is feasible for public health with relatively limited investment. The Wallace Center’s work described here can be replicated by other academic programs and Title V programs can partner with these programs. Using creative funding models—such as investing seed funding and matching funds with private partners—can create new training opportunities with relatively lower start-up funds. This requires reimagining academic collaboration with the private sector, including with entrepreneurial partners. It also requires transforming cross-academic partnerships to engage technology-driven departments. Building these new partnerships allows public health to leverage additional resources in partnership with technology and business. Diversifying project teams to include public health and technology perspectives also allows for organic training in MCH and innovation across trainees. Similarly, diversifying the modality of training—from applied courses to internship placements—expands the opportunities for training, partnership, and impact.

Technology is here to stay and its intersections with health and equity will continue to grow. To ensure future MCH leaders are prepared to serve at this intersection, we must expand MCH training to include applied knowledge and skills in technology and innovation so they are prepared to center the needs and voices of underserved populations, leverage novel research methods that are ethical and sound, and develop and evaluate innovative interventions in direct collaboration with a diverse range stakeholders.

## Data Availability

Not applicable.
